# Mental Health and Fall rates among older adults: Influence of exercise and unmet health care

**DOI:** 10.1093/geroni/igaf122.3052

**Published:** 2025-12-31

**Authors:** Idorenyin Udoh, Wah Wah Myint, Matthew Smith

**Affiliations:** University of Michigan Flint, Flint, Michigan, United States; Texas A&M University, College Station, College station, Texas, United States; Texas A&M University, College Station, Texas, United States

## Abstract

**Background:**

Physical activity and unmet health care (UNHC) are important factors influencing poor mental health and increased risk for falls among older adults. This study examines the impact of physical activity on UNHC needs and its subsequent effects on mental health outcomes and fall risk among community-dwelling older adults.

**Methods:**

Data were analyzed from 164,955 participants in the 2023 Behavior Risk Factor Surveillance System dataset. Two models were fitted: A multinomial logistic regression model with past-month poor mental health days (PMHD) (a three-categorical variable-i.e., 0 days, 1-13 days, 14-30 days) and a binary logistic regression model with past-year fall (a dummy variable, i.e., a fall or not). Both models adjusted for socio-demographics characteristics, physical activity, and UNHC.

**Results:**

Sixty percent of participants had no past-month PMHD, 26% reported 1-13 PMHD, and 14% reported 14-30 PMHD. Nearly 29% reported a fall -year fall. Participants with past-year fall were more likely to report 1-13 (RR = 1.96) and 14-30 (RR = 2.64) PMHD, respectively. Participants without physical activity limitations were less likely to report 1-13 (RR = 0.92) and 14-30 (RR = 0.49) PMHD, respectively. Individuals with UNHC were likely to report 1-13 (RR = 1.86) and 14-30 (RR = 3.33) PMHD, respectively. Participants with PMHD 1-13 (aOR=1.96) and 14-30 (aOR=2.64) were likely to report 1+ falls in the past-year fall, respectively. Those with UNHC were more likely to fall (aOR=1.63), while those who exercised were less likely to fall (aOR=0.69).

**Conclusions:**

Findings highlight the importance of physical activity and healthcare utilization in the prevention and interventions for poor mental health status and falls.

